# Extending letrozole dosing in patients with polycystic ovary syndrome undergoing ovulation induction with intrauterine insemination

**DOI:** 10.1016/j.xfre.2025.06.002

**Published:** 2025-06-16

**Authors:** Kara B. Fields, Sharrón L. Manuel, Anthony C. Leonard, Roshni Venkatesh, Michael A. Thomas, Emily G. Hurley

**Affiliations:** a Department of Obstetrics and Gynecology, University of Cincinnati, Cincinnati, Ohio; b Department of Biostatistics, Health Informatics, and Data Sciences, University of Cincinnati, Cincinnati, Ohio; c University of Cincinnati College of Medicine, Cincinnati, Ohio

**Keywords:** Ovulation induction, letrozole, polycystic ovary syndrome

## Abstract

**Objective:**

To determine the effect of prescribing a second course of letrozole within a single ovulation induction/intrauterine insemination (OI/IUI) cycle on pregnancy outcomes in patients with polycystic ovary syndrome (PCOS).

**Design:**

Retrospective cohort study.

**Subjects:**

Cases designated as OI/IUI in PCOS patients using letrozole alone for OI were identified. Patient (n = 183) and cycle (n = 405) demographics and outcomes were collected. Cycles in which an adequate response was achieved with a single course of letrozole (n = 333) were compared with cycles in which a second course was needed to achieve adequate follicular growth (n = 72). A second course was indicated in cases of suboptimal follicular growth after the initial course of letrozole. The relationships between single vs. a second course and pregnancy-related outcomes were examined adjusting for age and body mass index in logistic regressions.

**Exposure:**

Extended dosing with a second course of letrozole within an OI/IUI cycle in PCOS patients.

**Main Outcome Measures:**

Clinical pregnancy.

**Results:**

The clinical pregnancy rate was not significantly different for the extended-dose cycles (15.3%,11/72) compared with single-dose cycles (13.5%, 45/333) (odds ratio [95% confidence limit] = 1.15 [0.56–2.36]). After adjusting for age and body mass index, there remained no significant difference between groups.

**Conclusion:**

Extended dosing with a second course of letrozole in an OI/IUI cycle is associated with no significant difference in pregnancy and live birth outcomes. Thus, it is reasonable to give a second course of letrozole in a patient with PCOS, if indicated, which helps optimize the number of cycles needed to achieve pregnancy.

Ovulation induction with intrauterine insemination (OI/IUI) is a common first-line treatment for infertility related to ovulation dysfunction caused by polycystic ovary syndrome (PCOS). The OI cycle protocol consists of medication to stimulate follicular growth, usually achieved by clomiphene citrate (CC), or letrozole (LE), monitored via ultrasound. Letrozole is commonly used in cases of PCOS or CC resistance (1). In PCOS patients with anovulatory infertility, LE improves live birth and clinical pregnancy rates and reduces time-to-pregnancy compared with CC and is recommended as first-line treatment for OI (2, 3). There is evidence for LE decreasing risk of multiple gestation because of high monofollicular growth (4). However, in an assessment of clinical attitudes toward LE use in reproductive endocrinology practices, no standardized method of LE administration was revealed despite its widespread use among those surveyed (5). Thus, additional research regarding dosing strategies is warranted.

In clinical practice, initial LE dosing for OI/IUI cycles is often modest to reduce the risk of hyperstimulation and multiple gestation. Failure to respond to this initial dose is common and is managed by providing a second course of LE, considered “extended dosing” in this study, within the same cycle or with cycle cancellation, often with escalated dosing in subsequent cycles. Extended dosing with a second course of LE may result in a longer time to IUI within the cycle, may lead to increased costs, and there is a paucity of existing data regarding its effects on cycle outcomes.

Letrozole resistance has been defined as failure to ovulate after a 5-day regimen of LE (5 mg daily) for at least one OI cycle. Polycystic ovary syndrome patients with LE resistance were subsequently treated with longer durations of LE, including regimens of 7 and 10 days, depending on response, resulting in increased ovulation rates with the extended treatment (6). This suggests a strategy of escalating LE doses across multiple OI cycles but does not provide evidence for re-dosing within the first (same) cycle, nor does it provide evidence for IUI outcomes. Letrozole resistance in IUI cycles is more difficult to define, as ovulation is triggered with human chorionic gonadotropin (hCG) once follicles have matured. A study involving PCOS phenotypic subcategorization showed that all PCOS phenotypes responded to escalating doses of LE (7). It is well-established that increased doses of LE may be needed for OI in patients with anovulatory infertility related to PCOS, yet there is a paucity of evidence for extending, also referred to as re-dosing with a second course of LE, within a single OI/IUI cycle.

Infertility treatment involves challenging financial and emotional considerations, in addition to medical risks and uncertain outcomes. It is important to optimize treatment utility to avoid more costly and invasive assisted reproductive technologies. In theory, extended dosing with a second course of LE within an OI/IUI cycle when the initial response to the medication is poor helps to minimize the number of cycles needed to achieve pregnancy and possibly save money. Thus, the objective of this study was to determine whether this intervention has adverse effects on pregnancy outcomes.

## Materials and methods

### Study design

This study was a retrospective cohort analysis of 183 patients and 405 OI/IUI cycles performed between 2021 and 2023 at UC Health Center for Reproductive Health. Study approval was granted by the Institutional Review Board for the Protection of Human Subjects at the University of Cincinnati on 12/6/2021 (2021–0901). All data were deidentified on collection and patients were not asked to actively participate. All data were obtained from Epic electronic medical record system.

### Patients and OI/IUI protocol

Patients were between the ages of 20 and 42 and had an existing diagnosis of PCOS. Polycystic ovary syndrome was diagnosed according to the 2003 Rotterdam criteria. Per the 2018 International Evidence-Based Guideline for the Assessment and Management of PCOS, this is the most widely accepted criterion, with diagnosis requiring two of the following three metrics: irregular menses (<8/y); hyperandrogenism (clinical or biochemical); and polycystic ovaries on ultrasound (8). Patients completed OI/IUI using LE with hCG trigger. Patients began OI cycles with evaluation between cycle days 2–5 using transvaginal ultrasound to measure endometrial thickness and assess the ovaries. Endometrial measurements were taken per standard protocol (9). Patients proceeding with OI/IUI were treated with oral LE daily starting on day 3–5 of their menstrual cycle, for a total of 5–10 days. Letrozole dose ranged between 2.5 and 10 mg/day. Doses were chosen based on patient age, weight, anti-mullerian hormone (AMH) level, duration of infertility and previous unsuccessful OI cycles.

If suboptimal follicular growth was determined on the first monitoring ultrasound after the initial course of LE, a second dose of LE was given for an additional 5–7 days at the same or higher dose. In the clinic, suboptimal follicular growth, or LE resistance, is clinician-determined and usually is defined as no follicles >10 mm in size following LE dosing. The hCG trigger was administered in a single 250 mg subcutaneous injection to stimulate ovulation once follicles reached the appropriate size, approximately 20–21 mm in average diameter. Patients underwent IUI 24 or 36 hours after hCG trigger was administered according to standard of care. Timing was determined by each provider’s clinical decision-making based on follicular response and predicted growth from the time of ultrasound to the time of inducing ovulation. This decision was occasionally based on the availability of the patient or the clinic. It has previously been published that trigger timing of 24 vs. 36 hours for IUI cycles made no difference in clinical pregnancy rates of PCOS patients (10). Therefore, in our clinic, we have allowed the flexibility between these two trigger timings.

### Quantitative variables

The exposure variable was extended dosing with a second course of LE within a single OI/IUI cycle. Cycles using LE in combination with CC or gonadotropins were excluded, as well as cycles that were cancelled before IUI. Patients with history of cancer, chemotherapy, or radiation were also excluded, as were cycles that lacked follow-up information concerning whether or not pregnancy had been achieved.

The primary outcome observed was clinical pregnancy, defined as ultrasound-confirmed intrauterine pregnancy (IUP). Secondary outcomes were biochemical pregnancy, defined as a positive urine pregnancy test or quantitative hCG >5 mIU/ml, ectopic pregnancy, and live birth, defined as delivery after 24 weeks gestation. Delivery of singleton and multiple pregnancies, as well as loss to follow-up or transition of care were subgroups of the live birth outcome category.

### Data collection

Demographic characteristics of patients and cycle details were collected. Polycystic ovary syndrome patient characteristics included age, race, body mass index (BMI), AMH level, and concomitant presence of male factor infertility. Cycle characteristics included location and size of follicular growth, baseline endometrial thickness, endometrial thickness before hCG trigger, time interval from hCG trigger to IUI, semen preparation method, and total motile sperm count at time of IUI. If a cyst or follicle was measured in dimensions, the average diameter was recorded. When ultrasound was not performed on the day of hCG trigger, a predetermined value of 1 mm was added to the endometrial thickness per day between the ultrasound and the day of trigger, assuming typical growth patterns during stimulated cycles as previously observed (11).

### Statistical analysis

Single-dose and extended-dose cycles were compared on all baseline and outcome variables using *t*-tests for continuous variables and χ^2^ or Fisher’s exact tests on categorical variables. Anti-mullerian hormone and motile sperm counts were log-transformed before undergoing *t*-tests. Odds ratios (ORs) and 95% confidence limits were calculated to express the associations between extended-dose cycles and positive results on each of the three outcomes. Each of those three relationships was adjusted for age and BMI, using logistic regression, to obtain adjusted ORs. (Anti-mullerian hormone was considered for use as a covariate–see Results). Cycles were treated as independent observations, and the alpha for all tests was a two-tailed 0.05, unadjusted for multiple tests.

## Results

Participants included 183 patients who underwent a total of 405 cycles, with 1–8 cycles per patient, with a mean (SD) of 2.2 (1.3) cycles per patient. Mean (SD) age was 31.5 (4.4) years, and BMI was 32.9 (8.9). Nine cycles were excluded because of lack of pregnancy data.

A total of 405 cycles were analyzed, 333 of which involved a standard single-dose regimen of LE, and 72 that received extended dosing with a second course within the same cycle. Patient and OI/IUI cycle characteristics are reported in Table 1. On average, patients who were prescribed a second course were more likely to be younger (*P*<.001) and have a higher AMH level (*P*<.001). Race distribution was significantly different between groups, with 85% of the cycles in the single-dose group involving females of White race, vs. 74% in the extended-dose group (*P*=.02). Further subcategorization of race is listed in Table 1. Later cycle day of trigger and increased number of ultrasounds were inevitably significantly different in the extended-dose group. Follicle count on the day of hCG trigger was higher in the single-dose group than the extended-dose group (each *P*>.001). There were no differences between groups in BMI, presence of male factor infertility, endometrial lining measurement at baseline or at time of hCG trigger, laterality of follicular growth, total motile sperm count, or method of sperm preparation (fresh vs. frozen).

**Table 1:** Baseline characteristics.

Characteristic	Single-dose cycles (n = 333)	Extended-dose cycles (n = 72)	*P* value
Age (y)	32.3 (4.2)	30.0 (4.0)	<.001^a^
Body mass index (kg/m^2^)	32.8 (9.3)	33.8 (8.0)	.40
Race, n (%)			.02
White	284 (85)	53 (74)	
Black	3 (1)	4 (6)	
Hispanic	9 (3)	5 (7)	
Asian	23 (7)	7 (10)	
Unknown/other	14 (4%)	3 (4%)	
Presence of male factor infertility	93 (28%)	24 (33%)	.39
Anti-mullerian hormone level (ng/mL)	6.1 (5.0)	10.1 (11.2)	<.001^a^^,^^b^
Endometrial lining at baseline (mm)	4.8 (1.5)	4.8 (1.7)	.91
Endometrial lining at trigger (mm)	9.0 (1.7)	9.0 (1.8)	.82
No. of mature follicles at trigger	2.2 (1.2)	1.9 (1.0)	.05
Size of leading follicle at trigger (mm)	21.5 (1.8)	21.1 (1.9)	.12
Location of follicular growth			.08
Left	88 (26)	16 (22%)	
Right	101 (30%)	32 (44%)	
Bilateral	143 (43%)	24 (33%)	
Cycle day of trigger	12.6 (1.7)	16.7 (2.3)	<.001^a^
Total number of ultrasounds	2.2 (0.5)	3.3 (0.7)	<.001^a^
Total motile sperm count (×10^6^)	22.6 (20.9)	22.7 (26.6)	.69^b^
Semen preparation, n (%)			1.0
Fresh	301 (90)	65 (92)	
Frozen	32 (10)	6 (8)	

*Note:* Mean (SD) reported for continuous variables.

a Statistically significant *P* value <.05.

b *P* value from *t-*test on log-transformed values.

Missing data included 13 cycles without an associated AMH level, 8 cycles without endometrial lining measurements at baseline ultrasound, and 3 cycles without a documented cycle day of hCG trigger. There were single missing values for other baseline characteristics. Non-missing values are listed in Table 1, Table 2. In Table 2, the outcome of live delivery excluded one cycle without follow-up, and the adjusted ORs for the outcomes excluded one cycle with missing BMI.

**Table 2:** Primary and secondary outcomes.

Outcome	Single-dose cycles (n = 333)	Extended-dose cycles (n = 72)	OR (95% CI) favoring outcome with extended dose	*P*	OR (95% CI), adjusted for age and BMI	Adjusted *P*
Clinical pregnancy	45/333 (13.5%)	11/72 (15.3%)	1.15 (0.56, 2.36)	.71	0.95 (0.44–2.02)	.89
Any pregnancy	51/333 (15.3%)	14/72 (19.4%)	1.33 (0.69, 2.57)	.38	1.14 (0.57–2.26)	.72
Live birth	33/333 (9.9%)	8/71 (11.1%)	1.15 (0.51–2.62)	.67	0.87 (0.36–2.11)	.76
Singleton delivery	29	4				
Multiple delivery	4	4				
Loss to follow-up or transfer of care	0	1				
Pregnancy failure	12	2				

*Note:* BMI = body mass index; CI = confidence interval; OR = odds ratio.

Anti-mullerian hormone was considered for use as a covariate in the adjusted analyses, in addition to age and BMI. It was found to be not linearly associated with pregnancy rates, and furthermore was not associated in any simple U-shaped (quadradic) way, so it was not used in the adjusted analyses.

Outcome data are reported in Table 2. The incidence of a positive pregnancy test was not significantly different between cycles with standard single-dose LE regimens and cycles involving a second dose of LE, 51/333 (15.3%) vs. 14/72 (19.4%). Of the 51 single-dose LE cycles with a positive pregnancy test, 45 were ultrasound-confirmed IUP, and 6 were found to be either a biochemical pregnancy, pregnancy of unknown location, or ectopic pregnancy. Of the 14 extended-dose LE cycles that resulted in pregnancy, 11 were found to be clinical IUP, and 3 were either biochemical pregnancy, pregnancy of unknown location, or ectopic. These clinical IUP rates of 13.5% in the single-dose cycles vs.15.3% in the extended-dose cycles were not significantly different from each other (*P*=.71).

Adjusting for patient age and BMI had little effect on either the outcome of any pregnancy or of clinical IUP. The OR favoring any pregnancy for the extended-dose cycles went from 1.33 unadjusted to 1.14 adjusted, and for clinical IUP from 1.15 unadjusted to 0.95 adjusted.

Regarding live birth outcomes, there was no significant difference between groups. The incidence of live birth was 33/333 (10.0%) in the single-dose group and 8/72 (11.1%) in the extended-dose group. Of the 45 clinical IUPs in the single-dose group, there were 33 live births reported, 4 of which were multiples (12.2%). Of the 11 clinical IUPs in the extended-dose group, there were 8 live births reported, 4 of which were multiples (50.0%), and 1 found to be lost to follow-up or transfer of care without available records. After adjusting for age and BMI, there remained no difference in this secondary outcome across groups.

## Discussion

In this study, we evaluated various cycle outcomes after extending LE dosing with a second course within a single OI/IUI cycle in patients with PCOS. No significant difference in outcomes including positive pregnancy test, clinical pregnancy, or live birth was found between the cycles with and without a second course of LE. The extended-dose cycles did result in slightly higher unadjusted outcome rates, however, after adjustment, nearly identical rates in the two groups.

Extended-dose cycles demonstrated a later day of hCG injection administration compared with single-dose cycles, but the two cycle types had similar endometrial lining thicknesses at the time of trigger. Patients with higher AMH were more likely to be prescribed a second course, likely because of a more severe PCOS phenotype. Interestingly, extended dosing occurred more often in younger patients. These results are important in considering who may be an appropriate candidate for a second course of LE, in response to follicular growth on ultrasound.

There is limited literature on the use of additional doses and the use of higher doses of LE within an OI/IUI cycle, although this is frequently done in clinical practice when follicular growth is suboptimal after the initial course. Many studies have evaluated the effects of LE plus an additional agent, including dexamethasone, N-acetylcysteine, and CC in comparison to LE alone, but these did not involve dose analysis or regimen changes made within an OI/IUI cycle (12, 13, 14). A prospective randomized controlled trial comparing LE alone with LE plus gonadotropin for controlled ovarian stimulation used a step-up protocol for the LE-alone group: starting with 2.5 mg on cycle day 2–5 and increased by 2.5 mg per day for next 3 days (2.5 mg, 5 mg, 7.5 mg, 10 mg) (15). The escalating dose was applied to all patients in this group, rather than only in those who failed to respond to initial low-dose LE. Many studies have compared LE doses, including a randomized controlled trial which found no significant difference in pregnancy rates using 5 mg vs. 7.5 mg LE in women with PCOS undergoing OI, but there was no re-dosing during these trials (16). Furthermore, Mandelbaum et al. (17) found in their study that ovulation and pregnancy rates did not differ in PCOS patients requiring additional “stair-stepped” LE dosing compared with those not requiring additional doses of LE.

Our study aimed to isolate the effect of LE dosing by excluding cycles with gonadotropins. Furthermore, we specifically involved prescribing a second course of LE within a given OI/IUI cycle, rather than increasing doses across multiple cycles, because the goal of assisted reproductive technologies is to optimize the number of cycles needed to achieve pregnancy. Although specific dosing was not part of this analysis, the results of this study support the recent evidence for prolonging the duration of the initial LE course to 10 days to reduce time-to-pregnancy in OI cycles (17).

Limitations of this study include those inherent with a retrospective study design, missing data, and limited sample size. Missing data are described in the Results section, and primarily included AMH and baseline endometrial lining thickness. Specifically, the secondary outcome of live birth was limited by paucity of data because of loss to follow-up or transfer of care. Another limitation included measurements that were estimated by adding predetermined values when ultrasound was not performed on the exact day of hCG trigger, as described in the Materials and Methods. Perhaps the most substantial limitation, the sample size and subsequent power of this study, could be improved by expanding the data collection across a larger period of time or multiple institutions.

This analysis is further limited by the differences in baseline patient and cycle characteristics shown in Table 1. Of these differences in baseline characteristics, age and BMI were chosen for statistical adjustment, however with marginal change in the ORs of the relationships. Anti-mullerian hormone was initially considered for use as an adjustment factor, but was determined not to be useful as a covariate.

## Conclusion

In conclusion, this study provides evidence that extending dosing with a second course of LE in a single OI/IUI cycle is not associated with a significant difference in pregnancy outcomes. Thus, it is reasonable to prescribe a second course of LE in a patient with PCOS, which helps optimize the number of OI/IUI cycles needed to achieve a pregnancy and limit medical, emotional, and financial burden.

## CRediT Authorship Contribution Statement

**Kara B. Fields:** Writing – review & editing, Writing – original draft, Visualization, Methodology, Investigation, Data curation, Conceptualization. **Sharrón L. Manuel:** Writing – review & editing, Writing – original draft, Methodology, Investigation, Data curation. **Anthony C. Leonard:** Writing – review & editing, Validation, Software, Formal analysis. **Roshni Venkatesh:** Investigation, Data curation. **Michael A. Thomas:** Writing – review & editing, Supervision, Conceptualization. **Emily G. Hurley:** Writing – review & editing, Supervision, Project administration.

## Declaration of Interests

K.B.F. reports travel support from the University of Cincinnati Obstetrics & Gynecology Residency Program, outside the submitted work. S.L.M. has nothing to disclose. A.C.L. has nothing to disclose. R.V. has nothing to disclose. M.A.T. has nothing to disclose. E.G.H. has nothing to disclose.
